# Partitioning, repressing and derepressing: dynamic regulations in MLA immune receptor triggered defense signaling

**DOI:** 10.3389/fpls.2013.00396

**Published:** 2013-10-08

**Authors:** Cheng Chang, Ling Zhang, Qian-Hua Shen

**Affiliations:** ^1^State Key Laboratory of Plant Cell and Chromosome Engineering, Institute of Genetics and Developmental Biology, Chinese Academy of SciencesBeijing, China; ^2^Graduate University of Chinese Academy of SciencesBeijing, China

**Keywords:** plant NLRs, MLA, barley, cell death, immune signaling, transcription factors, transcription regulation

## Abstract

Plants and animals have evolved intracellular nucleotide-binding domain and leucine-rich repeat-containing immune receptors (NLRs) to perceive non-self and trigger immune responses. Plant NLRs detect strain-specific pathogen effectors and activate immune signaling leading to extensive transcriptional reprogramming and termination of pathogen infection. Here we review the recent findings in barley MLA immune receptor mediated immune responses against the barley powdery mildew fungus. We focus on nucleocytoplasmic partitioning of immune receptor, bifurcation of immune signaling, transcriptional repression and derepression connecting receptor activation to immune responses. We also discuss similar findings from other plant NLRs where appropriate.

## INTRODUCTION

Plants have evolved two major classes of immune receptors to detect non-self and defend themselves against pathogen infection (Jones and Dangl, 2006). The surface resident pattern recognition receptors (PRRs) mainly recognize conserved microbe-associated molecular patterns (MAMPs) while the intracellular nucleotide-binding and leucine-rich repeat receptors (NLRs) perceive strain-specific pathogen effectors that are delivered inside host cells (Zipfel, 2009; Dodds and Rathjen, 2010). Both PRR and NLR mediated cellular defense responses share an overlapping signaling network (Tsuda et al., 2009; Tsuda and Katagiri, 2010) but differ quantitatively and kinetically in nature (Tao et al., 2003; Caldo et al., 2004), nevertheless, NLR-triggered immunity is usually associated with rapid and localized host cell-death, termed hypersensitive reaction (HR), at the attempted pathogen infection sites (Shen and Schulze-Lefert, 2007; Boller and Felix, 2009; Maekawa et al., 2011b).

Plant NLRs are typically modular-structured, consisting of a central nucleotide-binding domain, C-terminal leucine-rich repeats, and a diversified N-terminal domain of either coiled-coil (CC) or TOLL/interleukin-1 receptor (TIR) subtype. The NLR receptors act as molecular switches to regulate immune responses by switching from an inactive form to an active form upon recognition of pathogen effector(s) and induced conformational changes from ADP- to ATP-bound state (Collier and Moffett, 2009; Lukasik and Takken, 2009; Takken and Goverse, 2012). The N-terminal CC or TIR domain may act as a signaling module for triggering host cell death (Swiderski et al., 2009; Krasileva et al., 2010; Bernoux et al., 2011; Collier et al., 2011; Maekawa et al., 2011a; Bai et al., 2012).

The barley MLA locus is highly polymorphic encoding a large number of allelic CC-subtype NLRs, each conferring isolate-specific disease resistance against the barley powdery mildew fungus, *Blumeria graminis* f. sp. *hordei *(*Bgh*; Seeholzer et al., 2010). The N-terminal CC domains of MLA are highly conserved in sequence (Seeholzer et al., 2010; Jordan et al., 2011), containing an EDVID motif shared with many other CC-subtype NLRs (Collier and Moffett, 2009). The more diversified C-terminal LRR region of MLA was shown to confer recognition specificity (Shen et al., 2003). Here we summarize our recent progresses towards understanding MLA-triggered immune signaling, emphasizing on receptor partitioning, signaling bifurcation, interacting transcription factors (TFs) linking receptor activation to defense response regulations. We also touch upon analogies in other plant NLR-mediated immune signaling pathways.

## DYNAMIC NUCLEOCYTOPLASMIC PARTITIONING OF MLA IMMUNE RECEPTORS

The barley intracellular MLA immune receptor has been shown to distribute between the nucleus and the cytoplasm (Shen et al., 2007). Using stable transgenic barley lines expressing a single copy of MLA1-HA fusion under the control of native 5′ regulatory sequences, fractionation experiments revealed that the majority of MLA1 is located in the cytoplasm and a small fraction (~5%) resides in the nucleus; and interestingly, its nuclear pool is increased upon inoculation of an incompatible *Bgh* isolate (Shen et al., 2007). Transient expression of a YFP-tagged natural MLA variant, MLA10, revealed that a MLA10-YFP fusion resides in both compartments in barley leaf epidermal cells (Shen et al., 2007; Bai et al., 2012). A mutation in the P-loop motif of MLA10 resulted in apparent increase of overall YFP signal intensity of MLA10-YFP in both compartments for unknown reasons (Bai et al., 2012), excluding the possibility that the P-loop motif of MLA10 is involved in nucleocytoplasmic partitioning. Similar nucleocytoplasmic distribution of the MLA10-YFP fusion was observed in the heterologous *N. benthamiana *system upon Agrobacterium-mediated transient expression and confocal imaging (Bai et al., 2012). Interestingly, similar nucleocytoplasmic partitioning of MLA1 was observed in *Arabidopsis* using a transgenic lines expressing MLA1-HA in a triple mutant background (Maekawa et al., 2012). Whether MLA immune receptors are regulated by conserved or distinct import/export machinery in these two plant species is currently unknown.

In recent years several plant NLR immune receptors have been shown to distribute between cytoplasm and nucleus (Deslandes et al., 2002; Burch-Smith et al., 2007; Shen et al., 2007; Wirthmueller et al., 2007; Cheng et al., 2009; Slootweg et al., 2010; Tameling et al., 2010; Hoser et al., 2013; Inoue et al., 2013; Ma et al., 2013). Some of them possess a canonical or predicted nuclear localization signal (NLS), for example the *Arabidopsis* RPS4/RRS1-R receptor pair and snc1, tobacco N and tomato I-2 resistance protein; while others, like MLA and potato Rx, do not harbor any discernible NLS signal. In this regard, it remains to be shown how the nucleocytoplasmic partitioning is regulated for most of these NLRs (Meier and Somers, 2011; Wirthmueller et al., 2013).

## BIFURCATION OF MLA-TRIGGERED CELL DEATH AND DISEASE RESISTANCE SIGNALING

**FIGURE 1:**
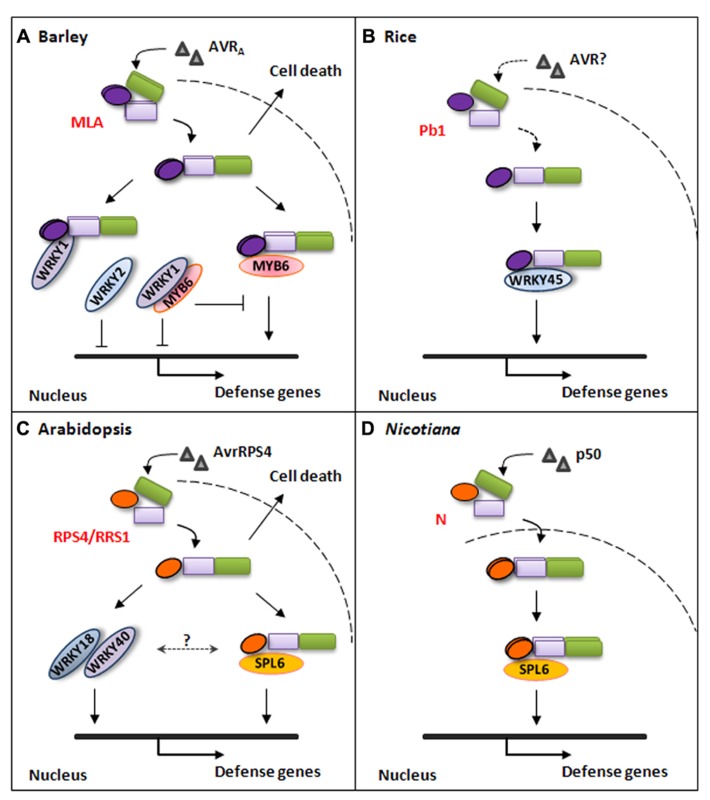
**Simplified models for plant NLR-triggered immune signaling pathways.** **(A)** Barley MLA immune receptor recognizes cognate AVR_A_ effector from *B. graminis* fungal pathogen and triggers disease resistance signaling in the nucleus or cell-death signaling in the cytoplasm. The activated MLA interacts with WRKY1 through its N-terminal CC domain to release MYB6 and by itself directly interacts with MYB6 to initiate defense gene expression. Barley WRKY1 and WRKY2 are repressors of defense responses. **(B)** Rice atypical NLR Pb1 interacts with WRKY45 to mediate immune responses against the rice blast fungal pathogen. The Pb1-WRKY45 association can prevent the TF from being degraded by the ubiquitin/proteasome system. **(C)** *Arabidopsis* NLR pair RPS4/RRS1 mediate disease resistance signaling against *Pst*DC3000(avrRPS4) through direct interaction with *At*-SPL6 or through WRKY18 and WRKY40 in the nucleus. RPS4 can also trigger cell-death signaling in the cytoplasm. **(D)** *Nicotiana* N immune receptor specifically recognizes a 50KD helicase domain (p50) from Tobacco mosaic virus (TMV) in the cytoplasm and activated N associates with SPL6 within distinct nuclear compartments to mediate immune responses against TMV.

Forced localization of MLA10 to either the cytoplasm or the nucleus, by adding either nuclear export signal (NES) or NLS to its C-terminus (CT), revealed distinct receptor activities in signaling (Shen et al., 2007; Bai et al., 2012). The nuclear pool of MLA10 is essential for powdery mildew disease resistance as transient expression of the MLA10-YFP-NES fusion, that is depleted from the nucleus, fails to restrict the growth of an avirulent *Bgh* isolate (Shen et al., 2007). Further, expression and enforced nuclear localization of the MLA10-NLS fusion revealed that the MLA nuclear pool alone is sufficient to confer disease resistance against *Bgh* in barley (Bai et al., 2012). Unexpectedly, upon transient expression in the heterologous *N. benthamiana* leaves, the MLA10-NES fusion was able to trigger markedly enhanced cell death signaling, whereas MLA10-NLS was unable to induce cell death (Bai et al., 2012). Although MLA10-triggered cell death in the heterologous *N. benthamiana* system is effector-independent, combined with functional analysis in barley these data strongly suggest a model for bifurcation of MLA signaling, in which MLA triggers cell death signaling in the cytoplasm but mediates disease resistance signaling in the nucleus, and these signaling activities of MLA can be uncoupled in a cell compartment-dependent manner (**Figure 1A**).

Signaling bifurcation was also shown for a TIR-type immune receptor, the *Arabidopsis* RPS4 (Heidrich et al., 2011), which recognizes the type III effector AvrRps4 secreted by *Pseudomonas syringae* (Gassmann et al., 1999) and triggers EDS1-dependent transcriptional reprogramming and disease resistance (García et al., 2010; Heidrich et al., 2011). RPS4 was detected in association with EDS1 in complexes in *Arabidopsis* or *N. benthamiana* upon coexpression (Bhattacharjee et al., 2011; Heidrich et al., 2011). AVR effector-dependent activation of RPS4 in *Arabidopsis* nuclei restricted* P. syringae* growth without inducing cell death, however, it triggered weak cell death if the cognate AVR was forced to localize in the cytoplasm (Heidrich et al., 2011). It was proposed that nuclear or cytoplasmic RPS4-EDS1 pools specify distinct subcellular defense signaling branches, and that coordinated action of both defense signals is required for full defense responses (García et al., 2010; Heidrich et al., 2011, Heidrich et al., 2012; **Figure 1C**).

Several recent reports have shown uncoupling of host cell death from disease resistance for both TIR- and CC-subtype NLR immune receptors (Coll et al., 2010; Heidrich et al., 2011; Bai et al., 2012; Sohn et al., 2012; Gao et al., 2013), together these add unambiguous evidence to support a model that for some NLRs HR-cell death and disease resistance are distinct but interconnected subcellular functions.

## MLA CC DOMAIN AS A PLATFORM FOR INTERACTING AND SIGNALING

MLA fragments harboring the N-terminal CC domain or other domains have been used for identifying MLA interactors in yeast two-hybrid screenings. The CC domain containing fragments identified the most MLA interactors, and interestingly, almost all of them interacted with the CC domain but not with the MLA full-length protein in further analysis in yeast and *in planta* upon transient coexpression (Shen et al., 2007; Chang et al., 2013; Chang and Shen, unpublished data), suggesting that the MLA CC domain alone can serve as a platform for interacting with or docking to signaling partners post MLA activation.

A crystal structure of MLA10 CC reveals that this domain can form a homodimer and this dimer configuration is shown to be critical for MLA activity (Maekawa et al., 2011a). In the heterologous *N. benthamiana* system, a role of the MLA10 CC domain in cell death signaling has been established by Agrobacterium-mediated transient expression (Maekawa et al., 2011a; Bai et al., 2012). MLA10 full-length protein triggered cell death requires an intact P-loop motif; and mutations in the MHD motif render MLA10 autoactive, triggering cell death in *N. benthamiana* and barley (Bai et al., 2012), together these findings point to a likely scenario in which MLA activation involves conformational changes driven by ATP-binding and hydrolysis cycles and releasing of the N-terminal CC domain, which adopts a homodimer conformation that could serve as a platform for signaling initiation (Maekawa et al., 2011a; Takken and Goverse, 2012). Since the MLA cytoplasmic pool alone is sufficient to trigger cell death we envisage the death signaling might first initiate from the cytoplasm and then transduced by as yet unknown signaling components.

## TRANSCRIPTION FACTORS AS DIRECT DOWNSTREAM COMPONENT IN MLA-ACTIVATED SIGNALING

Earlier studies thoroughly characterized the association between MLA and two barley WRKY TFs, WRKY1 and WRKY2 (Shen et al., 2007). WRKY1 and WRKY2 interact with the MLA CC domain but not with the full-length MLA protein in yeast, importantly, an AVR_A_ effector-dependent association of full-length MLA10 with WRKY2 was detected in the nucleus of barley cells using fluorescence life time imaging-fluorescence resonance energy transfer (FLIM-FRET) analysis (Shen et al., 2007). Barley WRKY1 and WRKY2 were demonstrated to act as repressors of basal immunity against the *Bgh* fungus in barley. It was hypothesized that MLA immune receptors target these WRKY repressors to derepress PAMP-triggered immunity thus potentiating defense responses (Shen et al., 2007; Shen and Schulze-Lefert, 2007).

Recently, we reported the identification of barley MYB6 as another MLA interactor (Chang et al., 2013). MYB6 interacts with the CC domain of MLA receptors, MLA1, MLA6 and MLA10, and interestingly MYB6 appears to specifically interact with the homodimeric form of the functional CC domain (Chang et al., 2013). Since the full-length MLA protein was unable to interact with MYB6 we interpret the association of MLA CC with MYB6 as event post MLA receptor activation, somewhat analogous to the interaction between MLA and WRKY1/2. Nevertheless, contrary to the WRKY1/2 repressor, MYB6 acts as a positive regulator in basal and MLA-triggered disease resistance against the powdery mildew fungus, demonstrated by virus-induced gene silencing (VIGS) and functional gene expression analysis in barley (Chang et al., 2013).

Since WRKY1/2 and MYB6 interact with the MLA CC domain, the potential interaction between WRKY1/2 and MYB6 was tested. Significantly, WRKY1, but not WRKY2, interacts with MYB6 and interferes with MYB6 DNA binding activity (Chang et al., 2013). It is noteworthy that barley WRKY1 and WRKY2 share the same domain structure and 72% sequence similarity, and their *Arabidopsis* homologues, *At*-WRKY18, *At*-WRKY40 and *At*-WRKY60, act redundantly as negative regulators in disease resistance against the bacterial pathogen *Pseudomonas syringae *pv.* tomato* DC3000 (*Pst*DC3000) and the *Arabidopsis*-infecting powdery mildew fungus* Golovinomyces orontii* (Xu et al., 2006; Shen et al., 2007; Pandey et al., 2010). However, surprisingly, it was recently reported that *At*-WRKY18 and *At*-WRKY40 are specifically required for mediating disease resistance against* Pst*DC3000 expressing effector AvrRPS4, shown by the specific susceptibility phenotype of the *wrky18 wrky40* mutant line infected with *Pst*DC3000(avrRPS4) but not with other tested *Pst*DC3000 strains (Schön et al., 2013). These findings indicate that WRKY18 and WRKY40 may function redundantly as positive regulators downstream of the RPS4/RRS1 pair, or alternatively that these WRKYs may be targeted and modified by AvrRPS4 which can be perceived by RPS4/RRS1, although the direct physical interaction between RPS4 and WRKY18 or WRKY40 was not detected in the presence or absence of the AVR effector (Schön et al., 2013; **Figure 1C**).

Several other TFs have recently been reported to function in NLR-mediated immune signaling (Inoue et al., 2013; Padmanabhan et al., 2013; **Figure 1**). The *Nicotiana* SPL6 TF was demonstrated to interact with the N immune receptor in subnuclear bodies once immune signaling is activated and SPL6 functions as a positive regulator in N-mediated immunity against Tobacco mosaic virus in *Nicotiana* plants (**Figure 1D**); Interestingly, like *At*-WRKY18 and *At*-WRKY40, the SPL6 paralog in *Arabidopsis* is also specifically required for RPS4-triggered disease resistance against *Pst*DC3000 (avrRPS4; Padmanabhan et al., 2013; **Figure 1C**). The rice WRKY45 was demonstrated to interact with Pb1, an CC-NB-LRR protein conferring panicle blast resistance in rice, and this interaction prevents ubiquitin/proteasome-mediated degradation of WRKY45, which is believed to be involved in Pb1-triggered blast resistance (Hayashi et al., 2010; Inoue et al., 2013; **Figure 1B**).

## REPRESSING AND DEREPRESSING: TRANSCRIPTIONAL REGULATIONS IN MLA-TRIGGERED IMMUNE SIGNALING

The R2R3-type MYB TF family members have undergone expansion in different plant lineages and are involved in regulating diverse biological processes (Stracke et al., 2001; Dubos et al., 2010; Feller et al., 2011; Raffaele and Rivas, 2013). One of the best characterized MYB TF is *Arabidopsis* *At*-MYB30 that plays a critical role in executing hypersensitive cell death in defense response to the bacterial pathogen *Xanthomonas* (Vailleau et al., 2002; Raffaele et al., 2008). Significantly, the transcriptional activity of *At*-MYB30 and resistance function is negatively regulated not only by the host protein *At*sPLA_2_-α through physical association in the nucleus (Froidure et al., 2010), but also by the *Xanthomonas* Type III effector XopD by relocalizing it to nuclear foci (Canonne et al., 2011).

Barley MYB6 is also a R2R3-type MYB TF that binds to the cognate *cis*-element *MBS I* and acts as a transcriptional activator to regulate gene expression (Chang et al., 2013). MYB6 activity in DNA-binding was evaluated in the presence of WRKY1 or MLA CC in electrophoretic mobility shift assay (EMSA) or *Arabidopsis* protoplast transfection assay. Interestingly, WRKY1 could suppress MYB6 DNA-binding activity, whereas the MLA10 CC domain markedly stimulated this activity, suggesting that MYB6 activity is antagonistically regulated by WRKY1 and MLA CC domain (Chang et al., 2013).

The tripartite interaction among WRKY1, MYB6 and MLA were dissected in details using yeast three-hybrid, *in planta* and *in vitro* protein interaction assays. It was demonstrated that the WRKY1-MYB6 association can be abrogated by the MLA10 CC domain in a WRKY1 CT-dependent manner, and subsequently MLA10 CC forms a complex with MYB6 in the nucleus. Importantly, MLA10 CC and an autoactive MLA10 full-length variant with a mutation in the MHD motif can antagonize WRKY1 suppression and markedly stimulates MYB6 DNA-binding activity, thus increases MYB6-dependent gene expressions in the *Arabidopsis* protoplast transfection system (Chang et al., 2013).

We propose a model in which WRKY1 repressor physically sequesters barley MYB6 from binding to the promoter of downstream target genes to prevent uncontrolled cell death and defense responses; upon perception of cognate effector activated MLA interacts with WRKY1 and releases MYB6 from suppression and stimulates its binding to cognate *cis*-acting elements to initiate disease resistance signaling (Chang et al., 2013; **Figure 1A**).

## CONCLUSIONS AND PERSPECTIVES

Data from barley MLA and other plant NLRs discussed here underlines the importance of nucleocytoplasmic trafficking and transcriptional regulation in plant NLR-mediated immune responses. Emerging evidence indicates that parallel mechanistics of regulation exist in mammalian NLR-mediated immunity. NLRC5 was recently presented as a transcription regulator to cooperate with TFs to induce MHC class I gene expression (Meissner et al., 2010, 2012), while CIITA was previously identified as a master transcription coactivator in regulating MHC class II gene expression (Ting and Davis, 2005); both NLRs shuttle between the cytosol and nucleus.

Specific and fundamental questions remain to be addressed to fill the gaps in MLA-activated immune signaling: what are the target genes commonly and distinctively regulated by WRKY1/2 and/or MYB6? How are MLA, WRKY1/2 and MYB6 regulated at post-translational level? What are the components/pathways involved in MLA-triggered cell death signaling in the cytoplasm? How does MLA regulate distinct immune activities in the nucleus and cytoplasm?

So far only a limited numbers of NLRs were shown to trigger defense signaling through direct association with TFs, which is likely downstream of AVR effector perception (**Figure 1**). Nevertheless, analogous mechanistics appears to be engaged with by both CC- and TIR-subtype of NLR receptors from either monocots or dicots to coordinate defense responses against diverse pathogens, including viral, bacterial and fungal pathogens (**Figure 1**). It is reasonable to envisage that NLRs are partially nuclear localized or translocated into the nucleus upon activation may orchestrate defense gene expression through transcriptional regulation. We are only at the beginning to unravel the dynamics of NLR-mediated signaling in the cytoplasm and the nucleus.
